# Enhanced calcium and thermal stability of whey protein hydrolysate stabilized emulsions by ultrasound-assisted glycosylation: Influence of the degree of glycosylation

**DOI:** 10.1016/j.ultsonch.2025.107413

**Published:** 2025-05-31

**Authors:** Congzhen Shi, Jun Liu, Yuanyuan Deng, Pengfei Zhou, Yan Zhang, Zhihao Zhao, Xiaojun Tang, Ping Li, Jiarui Zeng, Mingwei Zhang, Guang Liu

**Affiliations:** a Sericultural & Agri-Food Research Institute Guangdong Academy of Agricultural Sciences/Key Laboratory of Functional Foods, Ministry of Agriculture and Rural Affairs/Guangdong Key Laboratory of Agricultural Products Processing, Guangzhou 510610, China; b College of Food Science and Technology, Huazhong Agricultural University, Wuhan 430070, China; c Food Laboratory of Zhongyuan, Luohe 462300 Henan, China

**Keywords:** Whey protein hydrolysate, Ultrasound-assisted wet-heat glycosylation, Degree of glycosylation, Interfacial behavior, Emulsion, Thermal stability

## Abstract

Glycosylation can enhance the thermal stability and ionic tolerance of protein hydrolysate emulsions. Ultrasound-assisted wet-heat glycosylation (UA) and precise control over the degree of glycosylation (DG) are crucial for optimizing the modification effects. This study investigated the mechanisms underlying the influence of UA on the macroscopic stability of whey protein hydrolysate (WPH)-xylose (XL) conjugate emulsions. WPH-XL complexes (NWPH-XL) and wet-heat glycosylation (WH) served as controls. Both UA and WH reduced the interfacial adsorption capacity, interfacial interactions, and interfacial strength of WPH, with the extent of these reductions increasing with increasing DG. When DG up to 50 %, an “active” state characterized by high surface hydrophobicity and molecular flexibility, potentially promoting emulsion droplet aggregation during sterilization and storage. Notably, compared to WH, UA accelerated the reaction rate, inhibited the formation of AGEs, and improved the interfacial adsorption capacity, interfacial interactions, and interfacial strength of the conjugates by dissociating large aggregates. Consequently, UA conjugates with DG of about 20 % exhibited the strongest interfacial layer stability, effectively resisting oil droplet aggregation induced by 15 mM Ca^2+^ and sterilization at 121 °C (emulsion particle size of 276 nm). Taken together, these findings elucidated the mechanisms by which UA improves the stability of WPH-based complex emulsion systems from the perspective of interfacial behavior and highlighted the advantages of UA in enhancing the environmental tolerance of protein hydrolysates, expanding their potential applications in food emulsions.

## Graphical abstract

**Figure:**
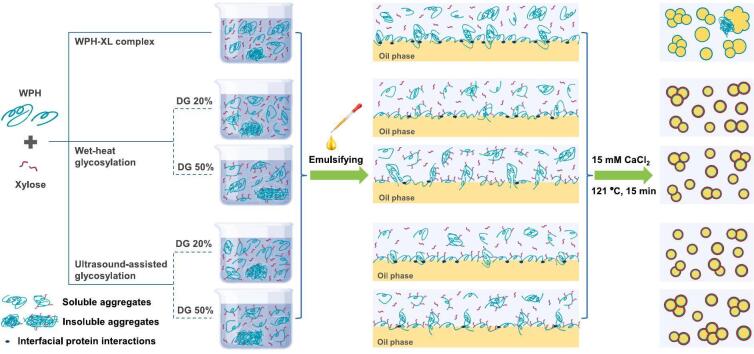

## 1 Introduction

Protein glycosylation modification based on the Maillard reaction (MR) can enhance protein steric hindrance and electrostatic repulsion, as well as regulate its amphiphilicity and spatial structure, thereby improving its functional properties [1]. Additionally, it can impart antioxidant and antibacterial properties [2], making it widely utilized in protein modification and processing [3]. To accelerate the reaction process, auxiliary techniques such as ultrasound and microwave are frequently employed [4], which can influence the structure and morphology of proteins [5]. Moreover, controlling the degree of glycosylation (DG) is crucial for regulating the number of saccharide grafts and, consequently, the structure and morphology of the modified proteins [6]. These factors ultimately lead to significant changes in the protein's interfacial adsorption capacity, interfacial layer stability, and, consequently, the macroscopic stability of the resulting emulsion.

Among these auxiliary techniques, ultrasound-assisted wet-heat glycosylation (UA) has demonstrated effectiveness in accelerating reaction rates and inhibiting protein aggregation, browning, and advanced glycation end product (AGE) formation [7]. Besides, previous studies have reported that glycosylation can heighten the interfacial layer stability [8,9] and emulsion stability of intact proteins or large-molecule peptides [10,11]. Regarding the effects of DG, previous studies [[12], [13], [14]] have reported that relatively high DG (rather than 30 %) can be beneficial for the interfacial properties and emulsion stability of proteins. However, these studies primarily focused on intact proteins or large-molecule peptides with well-preserved spatial structures and distinct hydrophilic-hydrophobic regions. In contrast, the effects of UA and DG on the interfacial and emulsifying properties of small-molecule peptides remain poorly understood, with a significant gap in in-depth and systematic research.

Protein hydrolysates with high degrees of hydrolysis (DH) contain small-molecule peptides that exhibit excellent digestibility, absorption properties, and diverse bioactivities [15], making them highly attractive for research and applications. However, their limited environmental tolerance, including sensitivity to high temperatures and metal ions (particularly divalent ions such as Ca^2+^), can readily induce aggregation, compromising their emulsification performance under conditions involving high temperatures or mineral additions. This significantly hinders their application in nutritional emulsions [16]. Among protein hydrolysates, whey protein hydrolysate (WPH) exhibits the best digestibility, absorption properties, and a wide range of applications, yet it is highly susceptible to high temperatures and metal ions [17]. Our previous study found that the effect of saccharide molecular weight on the glycosylation of small-molecule peptides is contrary to that observed with intact proteins or large-molecule peptides. Specifically, small-molecule saccharides, such as xylose (XL), are more suitable for glycosylation of WPH rich in small-molecule peptides compared to large-molecule saccharides [18]. However, the risk of AGE formation in these conjugates requires mitigation, and their calcium tolerance and long-term emulsion stability necessitate further improvement. While the regulation of UA and DG are anticipated to exert positive influences on these aspects, the interactive effects of UA and DG on the emulsion stability of glycosylated WPH remain elusive. Compared large-molecule peptide to intact proteins or large molecular peptides, the reduced size of small molecular peptides alters their intrinsic spatial structure and interfacial activity [17,19]. Theoretically, their functional properties are more sensitive to the effects of glycosylation [20]. Based on this, we hypothesize that the effects of UA and DG on the interfacial behavior and emulsion stability of WPH rich in small-molecule peptides differ from those observed with intact proteins or large-molecule peptides.

Therefore, this study employed small-molecule peptide-rich WPH and XL to investigate the effects of UA on the thermal stability in the presence of Ca^2+^ of WPH complex emulsion systems across varying DGs. It further explored the relationships between spatial structure, interfacial adsorption behavior, interfacial rheology, and macroscopic emulsion stability, ultimately elucidating the stabilization mechanism of glycosylated WPH complex emulsion systems from a multiscale perspective. Using WPH as a model for protein hydrolysates, this study aimed to provide novel insights into the effects of UA on the interfacial behavior and emulsion stability of protein hydrolysates in response to DG. This research endeavored to enhance the environmental tolerance of protein hydrolysates and facilitate their broader application in food emulsions.

## 2 Materials and methods

### 2.1 Materials

WPH (Hilmar™ 8350, with a DH of 12.5 %, 67.2 % of molecular weight (Mw) < 5000 Da, and 40.5 % of Mw < 1000 Da, containing 82.0 g protein/100 g, 3.0 g lactose/100 g, 5.0 g fat/100 g, 4.0 g moisture/100 g, and 5.0 g ash/100 g) was obtained from Hilmar Cheese Company (CA, USA). Xylose (XL) (S11037, 99 %) and o-Phthalaldehyde (OPA, S30210, ≥98 %) were purchased from Yuanye Biotechnology (Shanghai, China). Soybean oil (S110224), sodium dodecyl sulfate (SDS) (S108349, 99 %), 8-Anilino-1-naphthalenesulfonic acid ammonium salt (ANS-NH4, A151487, ≥95 %), and trichloroacetic acid (TCA, T104257, 99 %) were acquired from Aladdin Scientific (Shanghai, China). Pierce™ Dilution-Free™ Rapid Gold BCA Protein Assay, SeeBlue™ Plus2 Pre-Stained Protein Standard, and Pierce™ Glycoprotein Staining Kit were sourced from Thermo Fisher Scientific (Waltham, USA). Trypsin (1:250) was obtained from Solarbio Science & Technology (Beijing, China). Polystyrene microparticles (100 nm, 10 %) were purchased from Sigma-Aldrich (MA, USA). All other reagents were of analytical grade and were obtained from Sinopharm (Shanghai, China). Milli-Q ultrapure water (18.2 MΩ·cm) was used throughout the experiments.

### 2.2 Preparation of conjugates with varying DG via ultrasound-assisted and wet-heat glycosylation

Building upon the established method [18] in our previous research on this project, xylose was selected as the glycosylation saccharides due to its superior modification effect, and ultrasound-assisted wet-heat glycosylation (UA) was incorporated to optimize the reaction parameters for WPH-XL conjugates. The optimal conditions were determined through preliminary experiments to be a WPH concentration of 6 % (m/v), a WPH-to-XL mass ratio of 1:6, a pH of 10.0, a reaction temperature of 80 °C, and an ultrasound power of 500 W. The corresponding optimization data are presented in the supplementary material (Fig. S1). The WPH-XL mixture was prepared by dissolving the components in ultrapure water according to the specified proportions, followed by stirring at 600 rpm for 2 h and subsequent overnight storage at 4 °C to ensure adequate hydration. Upon returning to room temperature, the pH was adjusted to 10.0 using either 6 M NaOH or 6 M HCl, and the final WPH concentration was maintained at 6 %. The resulting mixture was then divided into 100 mL aliquots and transferred into sealed 100 mL reagent bottles for subsequent conjugate preparation.

UA was employed to prepare conjugates by varying reaction times (0.5, 1, 2, 4, 6, 8, 10, 15, 20, 25, 30, 40, 50, and 60 min). An ultrasonic homogenizer sonicator (JY99-IIDN, Scientz, Ningbo, China) equipped with a 15 mm probe and a magnetic stirring water bath was utilized. First, all conjugate samples were heated from RT to a preset temperature of 80°C in oil bath of 180 °C, and then maintained to a constant temperature water bath at 80 °C and 600 rpm of magnetic stirring. Sonication was conducted with 2-second pulses and 3-second interval. Subsequently, the reaction mixture was rapidly transferred to an ice bath to terminate the reaction. Wet-heat glycosylation (WH) conjugates were prepared under identical reaction parameters (temperature, stirring speed) but without ultrasound treatment, with reaction times of 2.5, 5, 10, 20, 30, 45, 60, 90, 120, 150, 180, 210, 240, 270, and 300 min, respectively. The DG of all conjugates was then determined. Reaction time-DG correlation curves were subsequently generated for both UA and WH conjugates. Conjugate samples with DGs approximating 10 %, 20 %, 30 %, 40 %, 50 %, and 55 % (with an error margin of less than 2 %) were selected and designated as UA10, UA20, UA30, UA40, UA50, UA55, and WH10, WH20, WH30, WH40, WH50, and WH55, respectively. Additionally, the following control samples were included: the original WPH solution (NWPH); a WPH and XL mixture solution without ultrasound or heating (NWPH-XL); and a WPH solution subjected to the same ultrasound and heating treatment (30 min) without XL (UHWPH). All glycosylated WPH samples were adjusted to pH 7.0 at room temperature and subsequently divided into aliquots and stored at −18 ℃.

### 2.3 Determination of the degree of glycosylation

The OPA reagent was freshly prepared, as previously described [21]. The conjugate solution was diluted with ultrapure water to a WPH concentration of 2.5 mg/mL. Subsequently, 200 μL of OPA reagent was added to 10 μL of the diluted conjugate solution. The mixture was then subjected to gentle shaking for 1 min, followed by incubation at 35 °C for 2 min. The absorbance at 340 nm was subsequently recorded using a Multiscan Spectrum (Infinite M200pro, Männedorf, Switzerland). NWPH-XL with an identical concentration served as the control. The free amino group (–NH_2_) concentration in the samples was determined by referencing an L-leucine standard curve (0–2 mg/mL). The DG was calculated using the following formula:

DG (%) = (C − c) / C × 100

where, C and c (mol/L) represent the free amino concentrations of NWPH-XL and conjugate samples, respectively.

### 2.4 Determination of the physicochemical properties of conjugates

#### 2.4.1 Browning intensity

The browning intensity of the conjugates and control samples was determined using the method described by Chen et al. [14]. Each sample was diluted with ultrapure water to achieve a final WPH concentration of 2.5 mg/mL. Subsequently, 200 μL of each diluted sample was transferred to a microplate, and the absorbance at 420 nm was measured using a Multiscan Spectrum spectrophotometer (Infinite M200pro, Männedorf, Switzerland). The recorded absorbance values were designated as A_420_.

#### 2.4.2 Turbidity

Based on a previously described method [22], the sample was diluted four-fold with ultrapure water to achieve a WPH concentration of 15 mg/mL. Turbidity was determined by measuring the absorbance at 600 nm using a Multiskan Spectrum (Infinite M200pro, Männedorf, Switzerland).

#### 2.4.3 Protein solubility

Samples were centrifuged at 10,000 × g for 20 min at ambient temperature. The supernatant was subsequently diluted 30-fold. Protein content within the supernatant was quantified using the Pierce™ Dilution-Free™ Rapid Gold BCA Protein Assay, with NWPH and equivalent glycosylated WPH (protein content determined via the Kjeldahl method) employed as the standard. The protein content of NWPH-XL was also determined. Protein solubility was calculated as the ratio of protein content in the conjugate supernatant to that in NWPH-XL.

### 2.5 Preparation of the emulsion

Following our previously published methodology [18] in our previous research on this project, the formulation and processing technology specific to foods for special medical purposes (FSMP) were taken into account in determining these parameters. 50 mL of conjugate solutions with different DG or control samples were combined with 3 g of soybean oil and diluted to a final volume of 75 mL with ultrapure water. This resulted in a 4 % (w/v) concentration of both WPH and soybean oil. A coarse emulsion was generated by subjecting the mixture to high-shear mixing at 10,000 rpm for 2 min using an Ultra-Turrax T25 homogenizer (Janke & Kunkel, Staufen, Germany). Subsequently, the coarse emulsion underwent two cycles of high-pressure homogenization at 80 % power (approximately 125–130 MPa) using a NanoGenizer 30 K homogenizer (Genizer, CA, USA). Finally, the emulsions were divided into three aliquots: (1) a fresh emulsion for immediate use, (2) a heat-sterilized emulsion (121 °C for 15 min), and (3) a calcium-fortified and heat-sterilized emulsion, that is supplemented with 15 mM CaCl_2_ (equivalent to 60 mg/100 mL calcium, a typical level found in emulsion-based foods) followed by sterilization at 121 °C for 15 min.

### 2.6 Determination of emulsion stability

To ensure an accurate and uniform evaluation of stability across all emulsion samples, each sample was subjected to vigorous mixing prior to testing. This step was necessary as some emulsions exhibited aggregation following sterilization. Consequently, all samples were vortexed at 2400 rpm for 2 min to achieve thorough homogenization.

#### 2.6.1 Particle size and ζ potential

Emulsion samples from Section 2.5 were subjected to a 400-fold dilution using phosphate-buffered saline (PBS, 20 mM, pH 7.0). Subsequently, Z-average particle size and zeta (ζ) potential of the diluted samples were determined using the automated program of a Zetasizer Nano ZSE (Malvern Instruments, Worcestershire, UK). The refractive indices of soybean oil and PBS were employed in the analysis, with values of 1.47 and 1.33, respectively.

#### 2.6.2 Backscattering (BS) intensity

Following the methodology described in the literature [23] with modifications, 0.04 wt% sodium azide (NaN_3_) was incorporated into the sterilized emulsion samples as outlined in Section 2.5 and thoroughly mixed. Subsequently, 3 mL aliquots of the emulsion were transferred to micro-sample bottles and stored at ambient temperature for a duration of 60 d. BS measurements were conducted using a single scanning program on a multiple light scattering spectrometer (Turbiscan Tower, Formulaction, Toulouse, France). The acquired scanning data were recorded, and average values were calculated for subsequent plotting of the BS curve.

### 2.7 Structure characterization of conjugates

#### 2.7.1 SDS-PAGE

Following the previously established protocol [18], identical loading and electrophoresis buffers were utilized. The molecular weight distribution of conjugates was determined by reducing SDS-PAGE employing a BeyoGel™ SDS–PAGE Precast Gel (Tris-Gly, 4–20 % gradient, Beyotime, Shanghai, China). Conjugates or control samples were diluted with ultrapure water to obtain WPH with a final concentration of 10 mg/mL and subsequently mixed with loading buffer in a 1:1 vol ratio. The mixtures were then denatured in a boiling water bath for 10 min. 15 μL of each sample and 10 μL of SeeBlue™ Plus2 Pre-Stained Protein Standard (3–198 kDa) as a molecular weight marker were loaded into separate wells. Electrophoresis was conducted at a constant voltage of 100 V, followed by standard staining, destaining, and gel imaging procedures.

#### 2.7.2 Intrinsic fluorescence spectroscopy

Following the methodology outlined in the literature [24], each sample was diluted with phosphate-buffered saline (10 mM, pH 7.0) to a working protein concentration of 0.25 mg/mL. Subsequently, intrinsic fluorescence spectra of the diluted conjugates and control samples were acquired using a fluorescence spectrophotometer (F-7000, Hitachi Co., Japan). Excitation was performed at 280 nm, and emission spectra were recorded within the range of 300–500 nm with a slit width of 5 nm.

#### 2.7.3 Surface hydrophobicity (H_0_)

The *H_0_* of conjugates and control samples was determined using the fluorescent probe ANS-NH_4_ [5]. Samples were diluted with PBS (10 mM, pH 7.0) to a series of concentrations (0.02, 0.05, 0.1, 0.2, 0.5, and 1 mg/mL). An 8 mM ANS-NH_4_ solution was prepared in the same PBS. Subsequently, 3 mL of each diluted sample was mixed with 30 μL of the ANS-NH_4_ solution. Fluorescence intensity was then measured using a fluorescence spectrophotometer (F-7000, Hitachi Co., Japan) with excitation at 390 nm and emission at 470 nm. *H_0_* was defined as the slope of the linear regression of fluorescence intensity against protein concentration.

#### 2.7.4 Protein molecule flexibility

Following the protocol outlined by Li et al. [25], the protein sample was initially diluted to a concentration of 1 mg/mL using a 50 mM Tris-HCl buffer solution at pH 8.0. Subsequently, a 1 mg/mL trypsin solution was prepared using the same buffer. A volume of 800 μL of the diluted sample was then mixed with 50 μL of the trypsin solution and incubated at 38 °C for a duration of 5 min. To terminate the enzymatic reaction, an equal volume of a 5 % (m/v) aqueous trichloroacetic acid (TCA) solution was added. The resulting reaction mixture was then centrifuged at 4000 g for 30 min at 4 °C. Finally, the absorbance of the supernatant at a wavelength of 280 nm was measured, a parameter often used as an indicator of protein molecule flexibility.

### 2.8 Characterization of interfacial proteins

#### 2.8.1 Composition of interfacial proteins

Following the previously described methodology [17] with appropriate modifications, proteins/peptides present at the oil–water interface of emulsions formulated with conjugates and NWPH-XL samples were isolated. Briefly, 1 mL of freshly prepared, unsterilized emulsion was transferred to a 1.5 mL microcentrifuge tube and subjected to centrifugation at 30,000 × g for 40 min at 20 °C. Subsequently, 700 μL of the lower aqueous phase was carefully withdrawn using a micro-syringe by piercing through the upper creaming layer. Then the remaining 300 μL of the emulsion was centrifuged again to completely withdraw the lower aqueous phase and combine it with the previous one, thereby achieving sufficient separation of the creaming layer and the aqueous phase. The final aqueous phase was then filtered through a 0.45 μm membrane and stored at −18 °C for subsequent analysis.

Following the two centrifugation steps, the fully-separated creaming layer was supplemented with 500 μL of ultrapure water. The mixture was subjected to continuous vortexing for 20 min, followed by centrifugation at 30,000 × g for 40 min at 20 °C. The precipitate and the lower aqueous phase were carefully removed using a micro-syringe to eliminate unadsorbed or weakly bound proteins/peptides. Subsequently, 960 μL of 0.1 % (m/v) SDS solution was added to the creaming layer to facilitate the re-dissolution of the proteins/peptides while maintaining the original oil phase concentration of the emulsion at 4 %. The mixture was then subjected to vigorous vortexing for 20 min. Finally, the extracted interfacial proteins were analyzed by gel electrophoresis as described in Section 2.7.1. Duplicate gels were prepared: one was stained with Coomassie Brilliant Blue, and the other was stained for glycoproteins using the Pierce™ Glycoprotein Staining Kit.

#### 2.8.2 Adsorbed protein concentration and interfacial protein coverage

The filtrates from Section 2.8.1 and the conjugates or NWPH-XL used for emulsion preparation were diluted 20-fold, and their protein/peptide concentrations were determined using the method described in Section 2.4.3. These measurements yielded the unadsorbed aqueous phase protein concentration (C_A_) and the total protein/peptide concentration (C_T_) in the original emulsion, respectively. The surface mean diameter D _(3,2)_ of the emulsion oil droplets was measured using a Mastersizer 2000 (Malvern, Worcestershire, UK) according to the method of Li et al. [17], the diluent liquid was 0.1 % SDS aqueous solution. The volume fraction of oil in the emulsion was denoted as Ф. The proportion of adsorbed proteins (AP) and the interfacial protein coverage (τ) were then calculated as follows:

AP (%) = (C_T_ − C_A_) / C_T_ × 100

τ (mg/m^2^) = (C_T_ − C_A_) × D _(3, 2)_ / (6 × Ф)

#### 2.8.3 Interfacial layer thickness

Building upon the previously described methodology [26] with some modifications, polystyrene microparticles (100 nm, 10 % wt) were diluted to a concentration of 0.1 % (wt) using PBS (10 mM, pH 7.0) that had been pre-filtered through a 0.45 μm membrane. Similarly, the conjugates and NWPH-XL were diluted in the same PBS solution to achieve a WPH concentration of 1 mg/mL, followed by filtration through a 0.45 μm membrane. Subsequently, 1 mL of the filtered solution was combined with 100 μL of the diluted polystyrene microparticle suspension and allowed to adsorb for a duration exceeding 30 min. The hydrodynamic diameters of both the adsorbed and unadsorbed polystyrene microparticles were then determined using a Zetasizer Nano ZSE (Malvern, Worcestershire, UK) operating under an automated program. Assuming refractive indices of 1.59 and 1.33 for the polystyrene latex particles and PBS, respectively, the viscosity of WPH system is 0.8972 cP at 25 °C. the interfacial layer thickness was calculated as the difference between the hydrodynamic diameters before and after adsorption.

### 2.9 Interfacial properties

Following the previously described methodology [27] with modifications, the interfacial properties of the conjugates and NWPH-XL were determined using an optical contact angle meter (OCA 25, DataPhysics Instruments GmbH, Germany). The original sample was diluted 60-fold with PBS (10 mM, pH 7.0) to achieve a WPH concentration of 1 mg/mL. Soybean oil was filled into a square glass cell tank to approximately three-quarters of its volume. The densities of the diluted samples and soybean oil were calculated and recorded by measuring their respective masses and volumes. All measurements were conducted at a constant temperature of 25 °C within an environment free from vibrational interference.

#### 2.9.1 Dynamic interfacial tension

A syringe containing 500 μL of the sample solution was mounted on the instrument. A needle with an outer diameter of 1.65 mm was inserted into the glass cell to approximately half its height. A 20 μL droplet was formed by precisely controlling the injection unit. The imaging system immediately commenced capturing images of the droplet's shape. Concurrently, the system software, SCA 20, analyzed the droplet shape according to the Young-Laplace equation, determining the interfacial tension (γ) with an accuracy of 0.01 mN/m. The interfacial tension was recorded as a function of adsorption time (t) at 5-second intervals for a duration of 180 min.

#### 2.9.2 Interfacial dilational rheology

Following the completion of the interfacial tension measurement, a frequency sweep test of interfacial dilational rheology was immediately conducted. The amplitude of the sinusoidal oscillations was 5 % (dA/A), with a frequency range spanning from 0.005 to 0.1 Hz. A total of six iterative steps were performed using logarithmic scanning. For each measurement, five sinusoidal cycles were executed, and a 200-second interval was maintained between successive frequency scans. Subsequently, the interfacial dilational modulus (E) was determined as a function of frequency, and the slope of the linear fitting curve obtained from a double logarithmic plot of the interfacial dilational modulus against frequency was calculated.

Following a frequency sweep, the oil–water interface was allowed to equilibrate for 4 min. Subsequently, an amplitude sweep test was conducted. The tests were performed sequentially and manually, employing amplitudes of 5 %, 10 %, 15 %, 20 %, 25 %, and 30 % (dA/A). The frequency was maintained at 0.02 Hz with a single iterative step and linear scanning. After each scan, the oil–water interface was again allowed to equilibrate for 4 min. For each measurement, five sinusoidal cycles were executed, and the corresponding changes in interfacial tension (γ) and surface area (A) were recorded during droplet expansion and contraction. The interfacial dilational modulus (E) was then calculated as a function of amplitude. Prior to each amplitude sweep, the interfacial tension (γ_0_) and surface area (A_0_) of the undeformed droplet were rapidly measured. Finally, a Lissajous curve was plotted with strain (ΔA/A_0_) against surface pressure (π = γ − γ_0_), where ΔA = A − A_0_.

### 2.10 Statistical analysis

All experiments or tests were conducted in triplicate unless otherwise noted. Experimental data were presented as mean ± standard deviation. One-way analysis of variance (ANOVA) was employed to assess statistical significance among samples using SPSS 20.0 software (IBM, USA). Duncan's multiple range test was subsequently applied to determine significant differences between groups at the *p* < 0.05 level.

## 3 Results and discussion

### 3.1 Effects of ultrasound treatment on the glycosylation reaction rate of WPH-XL

**Fig. 1:**
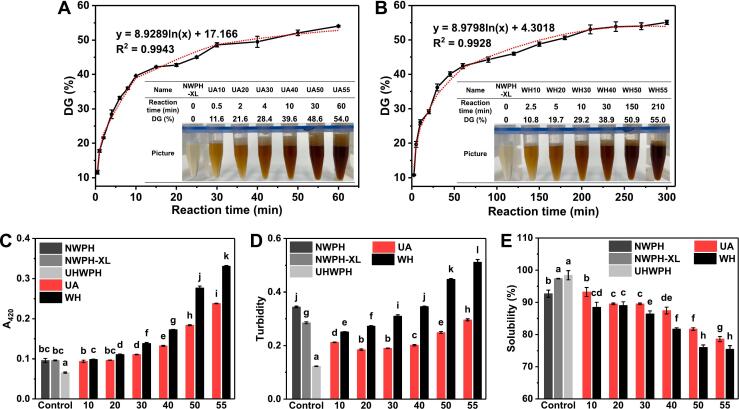
Correlation curves between reaction time and degree of glycosylation (DG) and prepared WPH-XL conjugates with DG of 10, 20, 30, 40, 50 and 55 % (±2%), A: ultrasound-assisted wet-heat glycosylation (UA), B: wet-heat glycosylation (WH). Browning intensity A_420_ (C), turbidity (D) and protein solubility (E) of WPH-XL conjugates with different DG prepared by UA and WH. NWPH refers to the untreated WPH solution, NWPH-XL refers to the WPH-XL mixture solution without ultrasound and heating treatment, and UHWPH refers to the WPH solution without XL addition but with the same ultrasound and heating treatment (30 min). Different letters on all columns indicate significant differences (*p* < 0.05).

Initially, the impact of ultrasound treatment on the glycosylation reaction rate of WPH-XL was investigated. As depicted in Fig. 1A and 1B, achieving the same DG required significantly less reaction time in the UA system compared to the WH method. The overall reaction rate of the UA system was 2.5–5 times faster than that of the WH system. While similar findings were reported by Mu et al. [12] and Chen et al. [7], with enhancement efficiencies reaching 20–30 times and 3–36 times, respectively, the present study exhibited a comparatively lower UA-induced rate enhancement, which can be attributed to the high degree of hydrolysis of the WPH used, leading to the exposure of more reactive groups and consequently higher inherent reactivity [20]. Furthermore, the strong reactivity of XL itself [28] contributed to a relatively high reaction rate under WH conditions.

### 3.2 Effects of UA on the physicochemical properties of conjugates with different DG

Maillard reaction (MR) typically lead to alterations in browning intensity, turbidity, and protein solubility, which can potentially impact the appearance, nutritional quality, and physical stability of emulsion systems. Hence, the impact of UA on these indicators was initially investigated.

Fig. 1C illustrates the changes in browning intensity (A_420_). As DG increased, the A_420_ values of both UA and WH conjugates gradually rose [24]. However, at the same DG, the A_420_ value of the UA conjugates was significantly lower (*p* < 0.05) than that of the WH conjugates, indicating that UA plays a beneficial role in mitigating browning intensity. This finding also suggests a reduction in the formation of AGEs, such as insoluble nitrogen-containing polymers (melanoidins) [29]. Furthermore, the decrease in browning intensity is advantageous for enhancing the color and overall appearance of emulsions. Similar findings, where ultrasound inhibited the increase in A_420_ values, have been reported by Chen et al. [14] and Dev et al. [24].

Evaluating turbidity changes can provide insights into the dispersion and aggregation state of proteins in solution [30]. As depicted in Fig. 1D, the turbidity of all UA conjugates was significantly lower (*p* < 0.05) than that of NWPH and NWPH-XL. Moreover, at identical DG, the turbidity of UA conjugates was substantially lower (*p* < 0.05) than that of WH. Intriguingly, the turbidity trend of UA conjugates with increasing DG differed markedly from that of WH. The turbidity of WH conjugates exhibited a continuous and significant increase (*p* < 0.05) with increasing DG. When DG was below 20 %, the turbidity of WH conjugates was lower than that of NWPH-XL, suggesting that a small degree of XL grafting could potentially disrupt protein aggregates [18]. However, with increased DG, prolonged heating during the MR induced protein aggregation and the formation of more insoluble polymers, resulting in a continuous rise in turbidity. Differently, the turbidity of UA conjugates initially decreased and subsequently increased with increasing DG. When DG was below 30 %, a continuous decrease in turbidity was observed. However, when DG exceeded 30 %, the disruption of aggregates facilitated by UA could no longer counteract the increased formation of insoluble polymers and heat-induced protein aggregation, leading to a gradual increase in turbidity [31]. These findings unequivocally demonstrate that UA plays a crucial role in inhibiting aggregate formation in glycosylated WPH [22], with a lower degree of aggregation observed at lower DG, which is expected to significantly influence its interfacial adsorption properties and, ultimately, emulsion stability.

Fig. 1E illustrates the changes in protein solubility. Compared to NWPH-XL, the solubility of all UA and WH conjugates exhibited a gradual decline with increasing DG, consistent with the A_420_ data. As DG increased, solubility gradually decreased, primarily attributed to the conversion of a greater proportion of glycoproteins into insoluble melanoidins during the later stages of MR [32]. In contrast, previous studies [7,12,33], have reported an increase in protein solubility following glycosylation, a phenomenon potentially linked to the relatively low solubility of the protein materials employed in those investigations. However, the WPH utilized in the present study exhibited a high initial solubility of approximately 95 %, leaving limited scope for further enhancement. As expected, the solubility of UA conjugates was significantly higher (*p* < 0.05) than that of WH conjugates across all DG values, indicating that UA effectively mitigated the decline in solubility of glycosylated WPH.

### 3.3 Effects of UA on the emulsion stability of conjugates with different DG

**Fig. 2:**
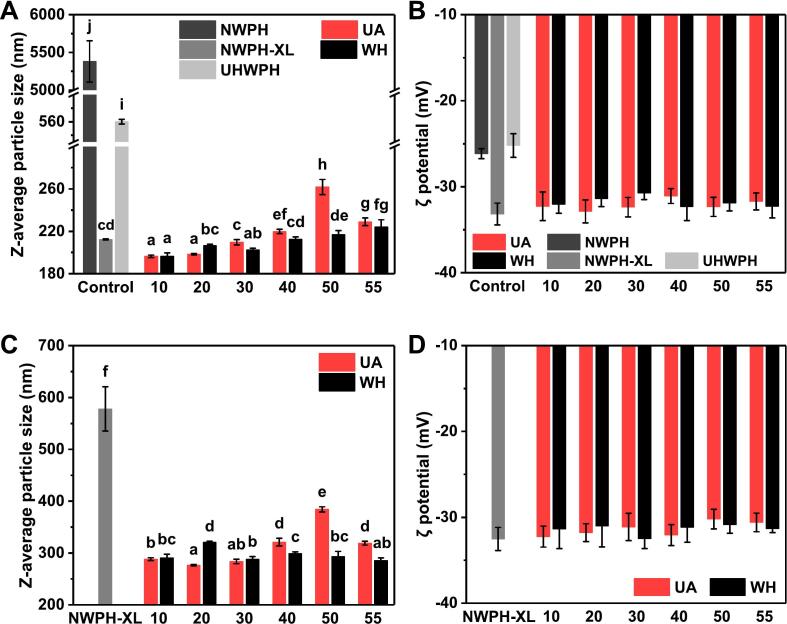
Average particle size and ζ potential of the sterilized emulsions of WPH-XL conjugates with different DG prepared by ultrasound-assisted wet-heat glycosylation (UA) and wet-heat glycosylation (WH), A & B: without CaCl_2_ addition, C & D: with 15 mM CaCl_2_ addition. The values 10–55 on the abscissa mean DG is 10 %–55 %. NWPH refers to the untreated WPH solution, NWPH-XL refers to the WPH-XL mixture solution without ultrasound and heating treatment, and UHWPH refers to the WPH solution without XL addition but with the same ultrasound and heating treatment (30 min). Different letters on all columns indicate significant differences (*p* < 0.05).

**Fig. 3:**
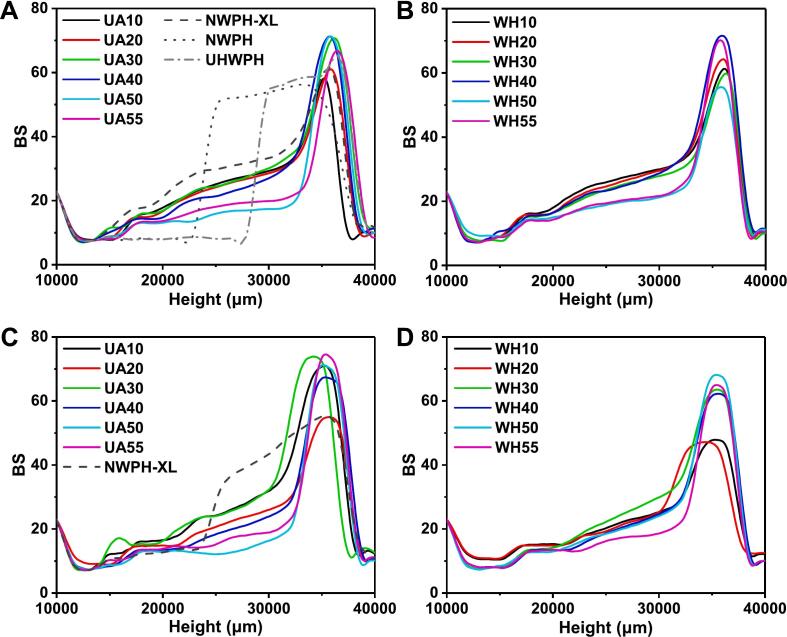
Backscattering intensity (BS) of sterilized emulsions of WPH-XL conjugates with different DG prepared by ultrasound-assisted wet-heat glycosylation (UA) and wet heat glycosylation (WH) after storage for 60 days, A & B: without CaCl_2_ addition, C & D: with 15 mM CaCl_2_ addition. The values 10–55 in the legend refer to DG 10 %–55 %. NWPH refers to the untreated WPH solution, NWPH-XL refers to the WPH-XL mixture solution without ultrasound and heating treatment, and UHWPH refers to the WPH solution without XL addition but with the same ultrasound and heating treatment (30 min).

Thermal stability and ionic tolerance are crucial parameters for assessing the industrial applicability of protein-based nutritional emulsions. Consequently, the influence of UA on the emulsion stability of conjugates with varying DG was evaluated following sterilization was performed both in the absence (Fig. 2A&B, 3A&B) and presence (Fig. 2C&D, 3C&D) of 15 mM CaCl_2_.

As illustrated in Fig. 2A, the emulsion particle size of NWPH-XL was significantly smaller (*p* < 0.01) than that of NWPH and UHWPH. This reduction can be attributed to the grafting of XL onto WPH during the MR under sterilization conditions, which enhances the thermal stability of the emulsion [34]. When the DG was below 40 %, the particle size of both UA and WH conjugate emulsions exhibited further reduction compared to NWPH-XL. Conversely, an opposite trend was observed when DG exceeded 40 %. Furthermore, the particle size of both UA and WH conjugate emulsions increased with increasing DG, suggesting that DG of about 20 % improved the thermal stability of the emulsions, whereas DG rather than 30 % had a detrimental effect. This observation contrasts with findings reported for the glycosylation modification of intact proteins or large-molecule peptides [14,35], highlighting the distinct nature of glycosylation modification in WPH, which is rich in small-molecule peptides. When DG was 20 %, the particle size of UA conjugate emulsions was significantly smaller (*p* < 0.05) than that of WH conjugates. However, when DG exceeded 20 %, the particle size of UA conjugates was generally larger than that of WH conjugates, indicating that the impact of UA on emulsion stability is also influenced by DG [7]. This phenomenon is likely related to variations in protein structure, aggregation degree, and interfacial behavior arising from the combined effects of DG and UA. Fig. 2B depicts the changes in emulsion ζ-potential. Compared to NWPH and UHWPH, the absolute values of ζ-potential for NWPH-XL and all conjugate emulsions exhibited a significant increase (*p* < 0.05), suggesting that glycosylation imparts stronger electrostatic repulsion, thereby contributing to the inhibition of protein and oil droplet aggregation [1]. However, no significant differences (*p* > 0.05) were observed in the ζ-potential of UA and WH conjugate emulsions across different DG values.

Fig. 2C illustrates the particle size of emulsions following the addition of 15 mM CaCl_2_ and subsequent sterilization. The particle size of all conjugate emulsions was significantly smaller (*p* < 0.01) than that of NWPH-XL, further corroborating the observation that glycosylation enhances the thermal stability in the presence of Ca^2+^ of WPH emulsions. With increasing DG, the particle size of UA conjugate emulsions exhibited an increasing trend, whereas the particle size of WH conjugates fluctuated slightly within a narrow range (285–320 nm). When DG was 20 %, the particle size of UA conjugates was significantly smaller (*p* < 0.05) than that of WH conjugates. However, when DG exceeded 30 %, the particle size of UA conjugates was significantly larger (*p* < 0.05) than that of WH conjugates. This observation suggests that UA conjugate emulsions with DG of about 20 % exhibit optimal thermal stability in the presence of Ca^2+^. Furthermore, as depicted in Fig. 2D, the ζ-potential values of NWPH-XL and all conjugate emulsions ranged from −30 to –33 mV, with no significant differences (*p* > 0.05) observed among them. These findings generally aligned with the trends observed for emulsions without the addition of CaCl_2_.

Emulsions undergo gradual and varying degrees of oil droplet aggregation and phase transition phenomena during storage [36]. To further assess the differences in emulsion stability after 60 d of storage, changes in BS values were measured. As depicted in Fig. 3, both UA and WH effectively mitigated oil droplet coalescence (manifested as a reduction in BS values), creaming (evidenced by a decrease in lower-layer BS values and a concomitant increase in upper-layer BS values), and phase separation (characterized by sharp changes in BS values between the upper and lower layers) of the WPH emulsion during storage [37]. However, with increasing DG, the extent of oil droplet aggregation and creaming in both UA and WH conjugate emulsions, with or without CaCl_2_, exhibited an increasing trend, indicating that elevated DG gradually diminished the storage stability of the emulsions. When DG was below 30 %, the BS values of UA conjugate emulsions were marginally higher than those of WH conjugates, suggesting that the storage stability of UA conjugate emulsions was slightly superior. Conversely, when DG exceeded 30 %, the opposite trend was observed, consistent with the particle size data.

### 3.4 The mechanism of UA on emulsion stability of conjugates with different DG

UA significantly influences the structural integrity of glycosylated WPH, impacting key parameters such as spatial conformation and molecular flexibility. These alterations, in turn, modulate the microscopic interfacial adsorption behavior and interfacial rheological properties of the glycosylated WPH at the oil–water interface. Consequently, the macroscopic stability of the resulting emulsions is significantly affected [38]. To elucidate the underlying mechanisms, this study focused on representative conjugate samples with DG of 20 %, 30 %, and 50 %. The investigation encompassed a multi-faceted approach, examining the impact of UA on the molecular structure, interfacial adsorption characteristics, and interfacial rheological properties of the glycosylated WPH complex emulsion system.

#### 3.4.1 Effects of UA on the structure of conjugates with different DG

**Fig. 4:**
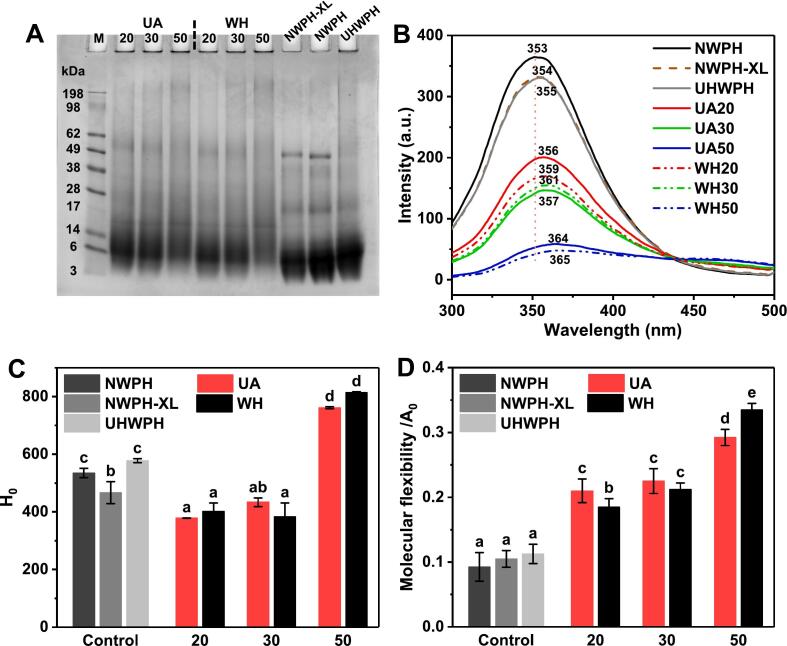
SDS-PAGE (A), intrinsic fluorescence spectra (B), surface hydrophobicity (H_0_) (C), and protein molecular flexibility (D) of WPH-XL conjugates with different DG prepared by ultrasound-assisted wet-heat glycosylation (UA) and wet-heat glycosylation (WH). The values 20–50 in the figure refer to DG of 20 %–50 %. NWPH refers to the untreated WPH solution, NWPH-XL refers to the WPH-XL mixture solution without ultrasound and heating treatment, and UHWPH refers to the WPH solution without XL addition but with the same ultrasound and heating treatment (30 min). Different letters on all columns indicate significant differences (*p* < 0.05).

First, the effects of UA and DG on the degree of XL grafting and the Mw of WPH were evaluated using reducing SDS-PAGE. As shown in Fig. 4A, compared to the three control samples, the bands of UA and WH conjugates in the region below 14 kDa were significantly diminished. Furthermore, the bands in the region above 14 kDa exhibited increased dispersion, continuity, and a discernible upward trend. This observation can be attributed to the increase in WPH Mw resulting from XL grafting, providing compelling evidence of XL conjugation with WPH [14]. Additionally, with increasing DG, the bands of UA and WH conjugates in the region below 14 kDa gradually weakened, while those above 14 kDa demonstrated an increasing trend. This observation was consistent with the decreasing solubility trend, suggesting that a higher DG correlates with a greater degree of XL grafting onto WPH. Similarly, a previous study [7] reported analogous findings for the glycosylation modification of peanut protein isolate and maltodextrin. Furthermore, at the same DG, the bands of UA conjugates in the region below 14 kDa were significantly more prominent than those of WH conjugates. This observation aligned with the finding that the turbidity of UA conjugates was significantly lower than that of WH conjugates. These results collectively suggest that ultrasound treatment effectively disintegrated large aggregates in glycosylated WPH into smaller aggregates [22,39]. In conclusion, both UA and DG exert significant influence on the spatial structure of the WPH system, leading to notable alterations in its hydrophilicity, hydrophobicity, and molecular flexibility.

Intrinsic fluorescence primarily reflects conformational changes in proteins/peptides by assessing microenvironmental alterations around tryptophan residues [24]. As depicted in Fig. 4B, compared to the three control samples, the fluorescence intensity of UA and WH conjugates decreased, and the maximum emission wavelength (λ_max_) exhibited a red shift. Moreover, as the DG increased, the fluorescence intensity of UA and WH conjugates decreased further, and the extent of the λ_max_ red shift gradually intensified. The decrease in fluorescence intensity and the red shift of λ_max_ can be attributed to an enhanced shielding effect on fluorescence and an increased polarity of the microenvironment surrounding tryptophan residues, respectively, both consequences of increased XL grafting [18]. Similarly, Li et al. [13] has shown that an increase in DG exerted a similar influence on the intrinsic fluorescence of glycosylated soy protein isolate (SPI), primarily indicating an increase in its internal or local structural flexibility. However, at the same DG, the fluorescence intensity of UA conjugates was higher than that of WH conjugates, which can be attributed to ultrasound treatment disrupting aggregates within the system [18]. The lower turbidity of UA conjugates compared to WH conjugates supports this observation. Interestingly, the λ_max_ of WH was higher than that of UA conjugates, consistent with the literature [14]. This discrepancy might be attributed to a higher degree of protein hydrolysis or unfolding under strongly alkaline conditions (pH 10) following prolonged heating, leading to greater exposure of tryptophan residues to a polar environment [24].

*H_0_* reflects the structural changes of proteins by evaluating the extent of hydrophobic group exposure on their surface [13]. As shown in Fig. 4C, at DG less than 30 %, the *H_0_* of UA and WH conjugates was significantly lower (*p* < 0.05) than that of the three control samples. This can be attributed to the lower degree of unfolding of WPH at low DG, where hydrophobic groups are not yet fully exposed on the molecular surface [24]. Furthermore, XL grafting inhibits the binding of 8-ANS to hydrophobic groups [12]. These two factors collectively contribute to the reduction in H_0_. However, when the DG increased to 50 %, the *H_0_* of UA and WH conjugates became significantly higher (*p* < 0.05) than that of the controls. At this point, WPH molecules were fully unfolded, exposing a greater number of hydrophobic groups on the surface [5,13]. Overall, the change pattern of H_0_ differs from that of intrinsic fluorescence because each emphasizes different aspects of protein structural alterations. Intrinsic fluorescence primarily reflects changes in the microenvironment of internal tryptophan, which is indicative of alterations in the looseness of the protein’s internal structure, whereas H_0_ reflects changes in the number of hydrophobic groups exposed on the protein surface. An excessive abundance of hydrophobic groups on the surface could promote the aggregation of emulsion oil droplets or proteins driven by hydrophobic interactions during sterilization and storage. Conversely, at lower DG, the reduction in surface hydrophobicity could yield contrasting effects.

Conformational changes in proteins inevitably influence their flexibility and capacity for structural rearrangement. Consequently, the differences in molecular flexibility were further investigated. As illustrated in Fig. 4D, the molecular flexibility of both UA and WH conjugates exhibited an increasing trend with increasing DG. Similarly, Li et al. [13] observed that the molecular flexibility of glycosylated SPI gradually increased with increasing DG. Interestingly, at a DG of 20 %, the lower degree of unfolding of WPH molecules and the presence of more aggregates resulted in lower molecular flexibility of WH conjugates compared to UA conjugates. As the DG increased to 50 %, prolonged heating in an alkaline environment led to greater protein unfolding (Fig. 4B & 4C) or hydrolysis of WH conjugates [24]. These factors gradually shifted the molecular flexibility of WH conjugates from a disadvantage to an advantage compared to UA conjugates. The increased molecular flexibility of glycosylated WPH suggests a reduction in its rigid structure, facilitating more facile structural rearrangements at the oil–water interface. Generally, enhanced protein molecular flexibility is advantageous for interfacial adsorption and emulsifying properties [25]. However, this “active” state with higher molecular flexibility may also promote the aggregation of emulsion oil droplets or proteins during sterilization and storage.

Based on the aforementioned analysis, it can be inferred that at DG of about 20 %, the grafting of a small amount of XL causes moderate unfolding of the UA conjugates, appropriately increasing molecular flexibility while maintaining a relatively stable structure with lower surface hydrophobicity. Consequently, the emulsion already exhibited excellent thermal stability in the presence of Ca^2+^ (Fig. 2). However, with increased DG, a greater amount of XL became grafted onto WPH, leading to more extensive protein unfolding. This exposed a larger number of hydrophobic groups, and the molecular flexibility and freedom for structural transformation of the protein molecules continued to increase. These changes, in turn, promoted the aggregation of emulsion oil droplets or proteins during Ca^2+^ addition, sterilization, and storage. Furthermore, these structural alterations have a critical impact on interfacial adsorption behavior and the stability of the interfacial layer. To elucidate the specific mechanisms by which UA and DG regulate the thermal stability in the presence of Ca^2+^ of the emulsion, interfacial adsorption and interfacial rheological properties were further analyzed in detail.

#### 3.4.2 Effects of UA on interfacial adsorption properties of conjugates with different DG

**Fig. 5:**
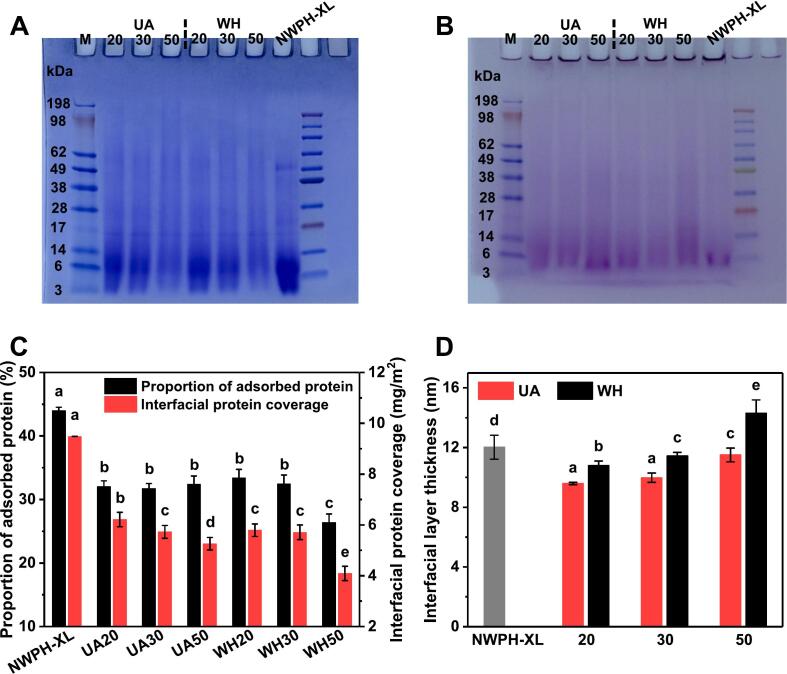
Interfacial protein composition (A: protein staining, B: glycoprotein staining), interfacial protein loading (C), and interfacial layer thickness (D) of WPH-XL conjugates with different DG prepared by ultrasound-assisted wet-heat glycosylation (UA) and wet-heat glycosylation (WH). The values 20–50 in the electropherograms and the abscissas refer to DG of 20 %–50 %. NWPH-XL refers to the WPH-XL mixture solution without ultrasound and heating treatment. Different letters on all columns indicate significant differences (*p* < 0.05).

Interfacial adsorption is a prerequisite for proteins to exhibit emulsifying properties [10]. Fig. 5A illustrates the differences in interfacial protein composition. The interfacial proteins of NWPH-XL were primarily concentrated in the region below 14 kDa. Compared to NWPH-XL, the band intensity in the region below 14 kDa for UA and WH conjugates was significantly diminished. As the DG increased, the band intensity in the region below 62 kDa gradually decreased, while faint macromolecular bands began to appear in the 62–198 kDa region. This observation suggests that glycosylation modification reduces the amount of protein adsorbed at the interface, particularly the fraction below 14 kDa [40]. In addition, the changes in glycoprotein composition at the interface were also evaluated (Fig. 5B). NWPH-XL exhibited weak glycoprotein bands, likely attributable to a small amount of non-covalent complexation between XL and WPH [41]. Compared to NWPH-XL, the glycoprotein bands of UA and WH conjugates gradually intensified with increasing DG, indicating that the amount of glycoprotein adsorbed at the interface increased as the DG increased.

As shown in Fig. 5C, further quantitative analysis of interfacial proteins revealed that both UA and WH conjugates significantly reduced interfacial protein content (*p* < 0.05), particularly interfacial protein coverage, which exhibited a clear decreasing trend with increasing DG. This observation can be attributed to the fact that XL grafting enhances the conformational flexibility and hydrophilicity of glycosylated WPH, leading to a propensity for detachment from the interface and migration into the aqueous phase, thereby reducing interfacial protein coverage [42]. Moreover, the interfacial protein coverage of WH conjugates was lower than that of UA conjugates, likely due to the larger aggregates in WH conjugates exhibiting weaker adsorption capacity [43]. These results were consistent with the findings on interfacial protein composition, collectively indicating that WH reduced the interfacial adsorption of WPH, with this reduction becoming more pronounced as DG increased. However, UA could enhance its interfacial adsorption by cleaving large aggregates.

In addition, interfacial layer thickness constitutes a crucial factor influencing emulsion stability [26]. As shown in Fig. 5D, compared to NWPH-XL, the interfacial layer thickness of all conjugates, except for WH with a DG of 50 %, was significantly reduced (*p* < 0.05). Moreover, at the same DG, the interfacial layer thickness of UA conjugates was significantly lower (*p* < 0.05) than that of WH conjugates. This phenomenon could be attributed to the dissociation of WPH aggregates by both UA and WH at lower DGs, leading to a reduction in interfacial layer thickness [43]. However, as the DG increased, the interfacial layer thickness of both UA and WH conjugates gradually increased, likely due to an increase in the molecular size of WPH following enhanced XL grafting and the formation of new aggregates during prolonged heating. This observation generally aligned with the trend observed for turbidity, which reflects the degree of WPH aggregation.

The alterations in the spatial structure, aggregation degree, and interfacial adsorption properties of the conjugates, induced by UA and DG, collectively influence interfacial protein interactions and interfacial viscoelasticity. These factors ultimately regulate the stability of the interfacial layer and the macroscopic characteristics of the emulsion. Therefore, accurate evaluation of their interfacial rheological properties is crucial.

#### 3.4.3 Effects of UA on interfacial tension and interfacial dilational rheological properties of conjugates with different DG

**Fig. 6:**
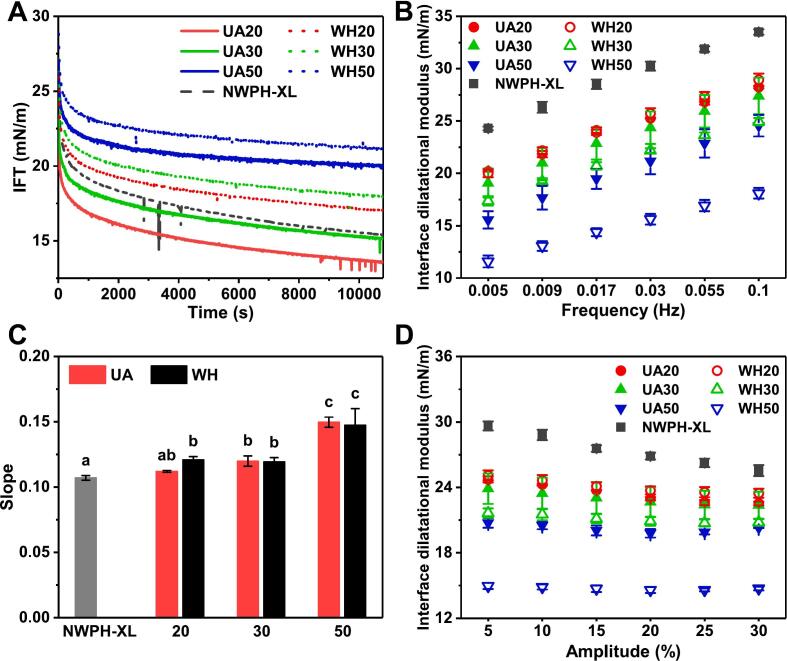
Dynamic interfacial tension (IFT) (A), interface dilatational modulus as a function of frequency (B), slope of a double logarithmic plot of the modulus versus frequency (C), and interface dilatational modulus as a function of amplitude (D) of WPH-XL conjugates with different DG prepared by ultrasound-assisted wet-heat glycosylation (UA) and wet heat glycosylation (WH). The values 20–50 in the legend refer to DG of 20%–50%. NWPH-XL refers to the WPH-XL mixture solution without ultrasound and heating treatment.

Fig. 6A shows the variation in interfacial tension with adsorption time. Compared to NWPH-XL, the interfacial tension of UA20 and UA30 conjugates decreased, while that of UA50 conjugates increased, and the interfacial tension of all WH conjugates increased. Furthermore, as DG increased, the interfacial tension of both UA and WH conjugates gradually increased. This phenomenon may be attributed to the relatively low molecular weight of WPH, rendering its interfacial activity more sensitive to changes in DG [20]. Excessive XL grafting can enhance the hydrophilicity of WPH, hindering its adsorption at the oil–water interface. At the same DG, the interfacial tension of UA conjugates was lower than that of WH conjugates, likely due to ultrasound treatment dissociate aggregates, making UA conjugates more prone to adsorb at the interface compared to WH conjugates [43]. This observation aligned with the results of interfacial adsorption properties. Overall, glycosylation enhances the conformational and molecular flexibility of WPH (Fig. 4), which inherently facilitates its interfacial adsorption and consequently reduces interfacial tension [36]. However, XL grafting could increase the hydrophilicity of numerous small-molecule peptides within WPH, resulting in a trend of increased interfacial tension. Nevertheless, UA exhibited a clear advantage in maintaining lower interfacial tension for glycosylated WPH, expected to exert a positive influence on interfacial rheological properties and interfacial layer stability.

To gain a deeper understanding of the mechanical properties and microstructure of the interfaces formed by UA and WH conjugates, interfacial dilational rheological properties were evaluated, including frequency and amplitude sweeps. Fig. 6B illustrates the variation of the interfacial dilational modulus with oscillation frequency. The interfacial dilational moduli of UA and WH conjugates were lower than that of NWPH-XL and decreased with increasing DG, indicating that glycosylation reduces the interfacial viscoelasticity and strength of WPH [27]. This is different from the results of reduced interfacial tension of UA20 and UA30, because the attached carbohydrate chains can also introduce steric hindrance and increase molecular flexibility, which may impede tight packing and strong intermolecular interactions between proteins at the interface. As a result, the interfacial film formed becomes less elastic and more prone to deformation, leading to a reduction in the dilational modulus. Furthermore, the moduli of UA conjugates tended to be higher than those of WH conjugates, suggesting that ultrasound treatment could mitigate the reduction in interfacial strength of WPH caused by glycosylation. Besides, the interfacial dilational modulus of all samples increased with increasing frequency. To further quantitatively analyze this frequency dependence, the slope values of the double-logarithmic linear fitting curves of the interfacial dilational modulus versus frequency were compared (Fig. 6C). The slope value of NWPH-XL was 0.11 (close to 0), indicating that its interface exhibited solid-like behavior dominated by elasticity and primarily dependent on interfacial protein interactions [44]. However, as DG increased, the slope values of UA and WH conjugates gradually increased, reaching approximately 0.15 at a DG of 50 %, indicating that glycosylation weakened the interfacial elastic behavior of WPH. Conversely, the exchange and structural reorganization behavior of these interfacial proteins between the interface and the bulk solution were enhanced [45]. This observation was consistent with the results of the conjugates' structure and interfacial adsorption properties.

Fig. 6D shows the variation of the interfacial dilational modulus with deformation amplitude. Excessive amplitude can influence the microstructure of the interface, providing valuable insights into its nonlinear interfacial rheology [45]. Firstly, the interfacial dilational modulus of NWPH-XL exhibited a significant decrease (*p* < 0.05) with increasing amplitude, indicative of strong interfacial protein interactions. However, following glycosylation modification, the modulus gradually decreased with increasing DG, and the amplitude dependence of the modulus also diminished progressively. This observation further suggests that higher DG values correlate with a greater reduction in interfacial protein interactions and interfacial strength [23]. Furthermore, the interfacial strength and interfacial protein interactions of UA conjugates were generally higher than those of WH conjugates.

**Fig. 7:**
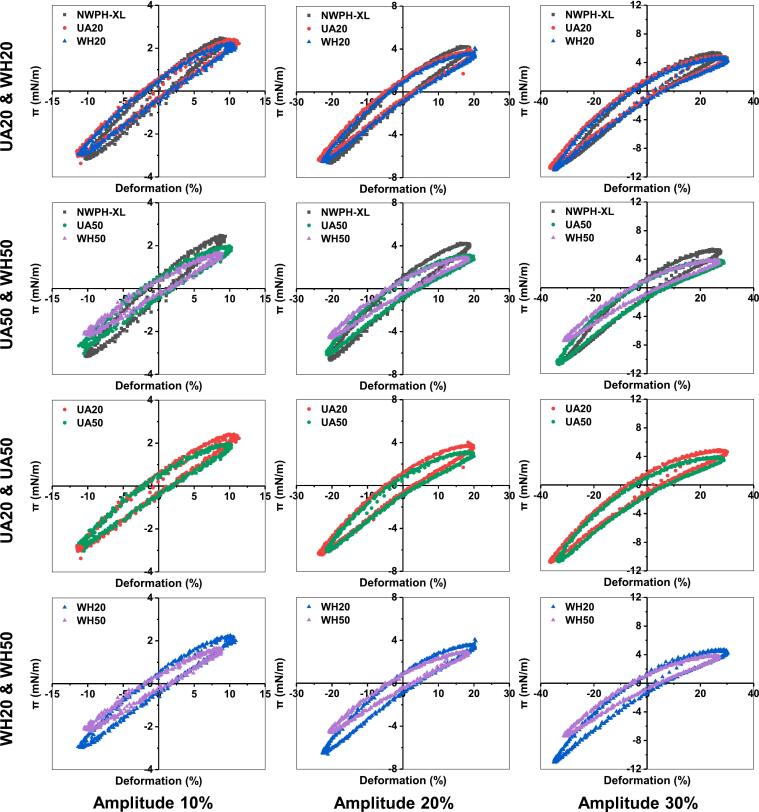
Lissajous curves obtained during amplitude sweeps of the oil–water interface stabilized by WPH-XL conjugates with different DG prepared by ultrasound-assisted wet-heat glycosylation (UA) and wet-heat glycosylation (WH). The values 20 and 50 in the legend refer to DG of 20 % and 50 %. NWPH-XL refers to the WPH-XL mixture solution without ultrasound and heating treatment.

Lissajous plots offer a more comprehensive assessment of interfacial behavior compared to dilational modulus, as they do not neglect nonlinear effects. As shown in Fig. 7, the Lissajous plots of all samples exhibited narrow elliptical shapes, indicating that their interfaces display predominantly elastic viscoelastic responses. As the deformation amplitude increased, the asymmetry of the Lissajous plots for all samples became more pronounced, suggesting a gradual transition of their interfaces toward nonlinear responses [23]. When the amplitude increased to 30 %, the rate of change in surface pressure during expansion for NWPH-XL gradually slowed (slope decreased), reflecting strain-softening behavior caused by the disruption of the interfacial microstructure (reduced interfacial protein density and weakened interactions). Conversely, during compression, the opposite trend was observed, indicating strain-hardening behavior, typically attributed to an increase in interfacial protein density [46]. Compared to NWPH-XL, the strain-softening behavior during expansion for UA20 and WH20 conjugates exhibited a slight enhancement, while the surface pressure corresponding to the maximum strain decreased slightly, maintaining a strain response behavior close to that of NWPH-XL. When the DG increased to 50 %, compared to NWPH-XL, the amplitude of the increase in strain-softening behavior during expansion and the decrease in the surface pressure corresponding to the maximum strain of UA and WH conjugates were further amplified. This observation indicates that excessive DG significantly reduced interfacial elasticity and increased viscosity, thereby diminishing the interfacial stiffness of WPH. Moreover, the surface pressure and slope corresponding to the maximum strain during compression, as well as the slope of the Lissajous plot for WH50, were significantly lower than those for UA50, indicating that the interfacial interactions, elasticity, and stiffness of UA conjugates were higher than those of WH conjugates [47]. Additionally, the effect of DG on the Lissajous plots was evaluated. Compared to UA20, the strain-softening behavior during expansion for UA50 conjugate was enhanced, and the surface pressure corresponding to the maximum strain decreased. Similarly, WH50 conjugate exhibited the same changes compared to WH20 but with a greater magnitude of change, and the slope of the Lissajous plot for WH50 was also significantly lower than that of WH20. These results further demonstrated that excessive DG reduced interfacial interactions, elasticity, and stiffness, and that the increase in DG caused more severe disruption to the interfacial microstructure of WH conjugates compared to UA conjugates [44]. These findings were consistent with the results obtained from interfacial dilational modulus measurements.

Overall, WPH constitutes a system of small-molecule protein peptides, which exhibit sensitivity to glycosylation modification. XL grafting tends to enhance its hydrophilicity and inhibit intermolecular interactions among WPH molecules. Furthermore, its conjugates exhibit an “active” state characterized by higher structural looseness and molecular flexibility. These factors promote protein exchange and structural reorganization between the interface and the bulk phase, ultimately leading to a reduction in interfacial protein adsorption and interactions, consequently weakening the viscoelasticity and stability of the interfacial layer. However, the introduction of ultrasound treatment can effectively cleave large aggregates within the conjugates, increasing interfacial protein adsorption and interactions. This, in turn, mitigates the reduction in interfacial strength and interfacial layer stability induced by glycosylation.

### 3.5 Mechanisms for maintaining stability

Previous studies have demonstrated that glycosylation can enhance the interfacial strength of proteins [8,9,48] and improve emulsion stability [10,11,21,24]. Moreover, analysis of research of Mu et al. [12], Li et al. [13] and Chen et al. [14] indicates that, within the degree of glycosylation (DG) range investigated, DG higher than 30 % show more obvious advantages in emulsion stability. These studies primarily focused on intact proteins or large-molecule peptides. However, the findings of this study present a contrasting observation. We observed that DG is below 20 % glycosylation could impart strong thermal stability in the presence of Ca^2+^ to WPH emulsions. However, glycosylation was found to diminish the interfacial layer stability of WPH, and the magnitude of this reduction increased with increasing DG, ultimately leading to a decrease in overall emulsion stability. These results suggest that WPH, which is rich in small-molecule peptides, exhibits distinct glycosylation effects compared to intact proteins and large-molecule peptides.

Specifically, glycosylation could increase steric hindrance and electrostatic repulsion between molecules within the WPH system, which was beneficial for inhibiting Ca^2+^ and heat-induced aggregation. Additionally, glycosylation caused relaxed spatial structure, increased surface hydrophobicity, and enhanced molecular flexibility of WPH, inherently improving its interfacial activity [25]. However, this “active” structural state could promote the aggregation of oil droplets or proteins during sterilization and storage. Furthermore, due to the high sensitivity of small peptides in WPH to glycosylation modification, XL grafting could enhance the hydrophilicity of WPH, ultimately reducing the interfacial protein coverage (especially of small peptides), while some glycoproteins were adsorbed at the interface. These changes weakened interfacial protein interactions, as well as interfacial elasticity and strength, ultimately leading to reduced interfacial layer stability. As the DG increased, more extensive XL grafting resulted in a continuous decrease in interfacial adsorption, interfacial interactions, and interfacial layer stability of the WPH system. In contrast, compared to WH, UA could break down aggregates within the glycosylated WPH system (as evidenced by its lower turbidity and interfacial layer thickness compared to WH), resulting in a higher protein or peptide content adsorbed at the interface in UA conjugates. This led to stronger interfacial interactions and elasticity, which were also beneficial for inhibiting Ca^2+^ and heat-induced aggregation. However, when the DG up to 50 %, the interfacial adsorption and interfacial interactions of both UA and WH conjugates decreased to extremely low levels. Consequently, the interfacial layer stability and the ability of the emulsion to resist aggregation become dominated by the interfacial layer thickness. Therefore, emulsions stabilized by WH conjugates with DG less than 40 % exhibited greater stability due to their thicker interfacial layers (Fig. 2, Fig. 3). In general, controlling DG to be less than or equal to 20 % was advantageous, while DG higher than 30 % was detrimental to emulsion stability. This conclusion suggests that combining ultrasound-assisted preparation with DG of about 20 % glycosylated WPH could not only significantly reduce preparation time, AGEs formation, and protein aggregation but also maintain optimal interfacial adsorption properties and interfacial layer stability, while simultaneously enhancing the thermal stability in the presence of Ca^2+^ of the emulsion.

## 4 Conclusion

In this study, WPH-XL conjugates with varying DG ranging from 10 % to 55 % were prepared using both UA and WH, respectively. The physicochemical properties of the conjugates and the thermal stability in the presence of Ca^2+^ of their corresponding emulsions were comprehensively compared. The effects of UA on the overall stability of glycosylated WPH emulsions with different DGs were elucidated from three perspectives: structural characterization, interfacial adsorption, and interfacial rheology. The results demonstrated that ultrasound accelerated the glycosylation rate of WPH and XL while simultaneously inhibiting the formation of AGEs and aggregates, as well as mitigating the decline in solubility. Compared to WH, UA enhanced the interfacial adsorption properties and interfacial interactions of the WPH system by effectively cleaving large aggregates, thereby improving its interfacial elasticity and strength. Furthermore, as the DG increased, more extensive XL grafting led to reduced interfacial adsorption and interfacial interactions of WPH, resulting in decreased interfacial elasticity and strength and, consequently, diminished overall emulsion stability. Additionally, DG of about 50 % induced an “active” state characterized by high surface hydrophobicity and molecular flexibility, which may promote the aggregation of oil droplets during sterilization and storage. Emulsions stabilized by UA conjugates with DG of about 20 % exhibited optimal interfacial layer stability and thermal stability in the presence of Ca^2+^. This study provides more precise insights into the effects of ultrasound-assisted and DG on the glycosylation of protein hydrolysates. Moreover, it establishes a feasible strategy for improving the environmental tolerance of protein hydrolysates and promoting their wider application in nutritional emulsions.

## CRediT authorship contribution statement

**Congzhen Shi:** Writing – review & editing, Writing – original draft, Software, Methodology, Investigation, Formal analysis, Conceptualization. **Jun Liu:** Software. **Yuanyuan Deng:** Visualization, Project administration. **Pengfei Zhou:** Methodology, Data curation. **Yan Zhang:** Validation, Supervision. **Zhihao Zhao:** Writing – review & editing, Software. **Xiaojun Tang:** Supervision. **Ping Li:** Data curation. **Jiarui Zeng:** Software, Methodology. **Mingwei Zhang:** Resources, Funding acquisition, Conceptualization. **Guang Liu:** Writing – review & editing, Funding acquisition, Formal analysis, Conceptualization.

## Declaration of competing interest

The authors declare that they have no known competing financial interests or personal relationships that could have appeared to influence the work reported in this paper.
