# Supplementary material for: Enhanced calcium and thermal stability of whey protein hydrolysate stabilized emulsions by ultrasound-assisted glycosylation: Influence of the degree of glycosylation
Source: Ultrason Sonochem. 2025 May 31;119:107413. doi: 10.1016/j.ultsonch.2025.107413 (PMC12171524; doi:10.1016/j.ultsonch.2025.107413)
Supplement: Supplementary Data 1 [file mmc1.docx]

**Supplementary material**

Fig. S1. Effects of ultrasonic power (A-C), reaction temperature (D-F), pH (G-I), ratio of WPH to XL (J-L) and WPH concentration (M-O) on the glycosylation reaction of WPH with XL. WPH: whey protein hydrolysate; XL: xylose; DG refers to the degree of glycosylation; A_294_ and A_420_ refer to the browning intensity at wavelengths of 294 nm and 420 nm, respectively. Different letters on the same line with same color indicate significant differences (*p* < 0.05).
